# Genome analysis of Excretory/Secretory proteins in *Taenia solium* reveals their Abundance of Antigenic Regions (AAR)

**DOI:** 10.1038/srep09683

**Published:** 2015-05-19

**Authors:** Sandra Gomez, Laura Adalid-Peralta, Hector Palafox-Fonseca, Vito Adrian Cantu-Robles, Xavier Soberón, Edda Sciutto, Gladis Fragoso, Raúl J. Bobes, Juan P. Laclette, Luis del Pozo Yauner, Adrián Ochoa-Leyva

**Affiliations:** 1 Instituto Nacional de Neurología y Neurocirugía, México, D.F., C.P. 14269, México; 2 Unidad Periférica del Instituto de Investigaciones Biomédicas en el Instituto Nacional de Neurología y Neurocirugía, México, D.F., C.P. 14269, México; 3 Instituto Nacional de Medicina Genómica, Periférico Sur No. 4809, Col. Arenal Tepepan, Delegación Tlalpan, México, D.F. C.P. 14610, México; 4 Instituto de Biotecnología, Universidad Nacional Autónoma de México, Avenida Universidad 2001, Cuernavaca, Morelos, C.P. 62210, México; 5 Instituto de Investigaciones Biomédicas, Universidad Nacional Autónoma de México, México, D.F., C.P. 04510, México; 6 Unidad de Genómica de Poblaciones Aplicada a la Salud, Facultad de Química, UNAM-Instituto Nacional de Medicina Genómica (INMEGEN), Periférico Sur No. 4809, Col. Arenal Tepepan, Delegación Tlalpan México, D.F. C.P. 14610, México

## Abstract

Excretory/Secretory (ES) proteins play an important role in the host-parasite interactions. Experimental identification of ES proteins is time-consuming and expensive. Alternative bioinformatics approaches are cost-effective and can be used to prioritize the experimental analysis of therapeutic targets for parasitic diseases. Here we predicted and functionally annotated the ES proteins in *T. solium* genome using an integration of bioinformatics tools. Additionally, we developed a novel measurement to evaluate the potential antigenicity of *T. solium* secretome using sequence length and number of antigenic regions of ES proteins. This measurement was formalized as the Abundance of Antigenic Regions (AAR) value. AAR value for secretome showed a similar value to that obtained for a set of experimentally determined antigenic proteins and was different to the calculated value for the non-ES proteins of *T. solium* genome. Furthermore, we calculated the AAR values for known helminth secretomes and they were similar to that obtained for *T. solium*. The results reveal the utility of AAR value as a novel genomic measurement to evaluate the potential antigenicity of secretomes. This comprehensive analysis of *T. solium* secretome provides functional information for future experimental studies, including the identification of novel ES proteins of therapeutic, diagnosis and immunological interest.

The secretome refers to the set of proteins that are excreted/secreted by a given cell, including extracellular-matrix (ECM) proteins, vesicle proteins (e.g., from microsomal vesicles) and proteins shed from the cell membrane^1^. These Excretory/Secretory (ES) proteins play important roles in development, adhesion, proteolysis and extracellular matrix organization of the organism. In parasitic organisms, the ES proteins play important roles acting as virulence factors and as immune regulators to control the host immune recognition during infection. The ES proteins are crucial for parasite survival inside and outside the host and their expression usually changes in response to several environmental stimuli^1^. As the ES proteins are involved in clinical manifestations of the host organism, they represent attractive drug targets for the development of novel therapeutic strategies^2^. Moreover, ES proteins are an important source of immunogenic proteins due to their accessibility to be recognized by the host immune system. Thus, considerable attention has been made in ES proteins as biomarkers to detect the presence of a parasite and/or the status of the infection in different infectious diseases^3^,^4^,^5^,^6^. The prediction of ES proteins from sequenced genomes is a novel strategy used to prioritize the experimental study of new therapeutic and immunodiagnostic targets for human parasitic diseases^2^. The ability to sequence the whole genome of parasite organisms provides the opportunity to *in silico* screen for the encoded secretomes and for the most probable antigenic proteins before undertaking confirmatory experiments. The increasing availability of genomes provides the opportunity to systematically examine their encoded secretomes using bioinformatics approaches.

Echinococcosis (hydatid disease) and cysticercosis caused by the proliferation of larval tapeworms in vital organs, are important neglected tropical diseases^7^. Cysticercosis is a tissue infection caused by the *Taenia solium* parasite (known as the pork tapeworm). The life cycle includes pig as intermediary host and human as definitive host. The tapeworm is the adult stage of *T. solium* parasite and infects the human intestine delivering the eggs into the human feces. The intermediary host becomes infected by ingesting contaminated vegetation with eggs and subsequently oncospheres hatch, penetrate intestinal wall and circulate to musculature. The oncospheres develop into larval stage (cysticerci) in muscle and central nervous system (CNS). The life cycle is completed when humans ingest raw or undercooked infected meat and develop the adult tapeworm in the intestine^8^,^9^. However, humans accidentally ingest the eggs and develop the cysticerci. In humans the cysticerci is predominantly established in the CNS causing neurocysticercosis (NC), which is the most common worldwide tapeworm infection of the brain and it is an endemic disease of developing countries^10^,^11^. The NC causes symptoms that range from cephalea and dizziness to epilepsy and severe intracranial hypertension, impacting on the social and economic development of the affected communities^11^,^12^.

Tapeworms (Platyhelminthes, Cestoda) secrete several ES molecules to regulate the host immune system for parasite survival^13^,^14^,^15^,^16^,^17^,^18^,^19^. ES proteins involved in the uptake and sequestration of host hydrophobic molecules^20^ and mediating the host immune response to parasite infection^21^ have been experimentally characterized in different life cycle stages of *T. solium*^22^. Also, several ES proteins with peptidase activities has been reported^23^,^24^,^25^. However, since no curated protein database and no genome sequence for *T. solium* was then available, those studies only produced partial lists of Excreted/Secreted proteins. Recently, the *T. solium* genome has been published^26^, allowing us the opportunity to characterize the ES proteins encoded in the genome and to *in silico* screen for the most probable protective antigens before undertaking confirmatory experiments. The prediction of number of antigenic regions per each protein at genome-wide level can help in the design of vaccine components and immunodiagnostic reagents. There are many bioinformatics methods to predict antigenic regions from a protein sequence. The classical approach of epitope prediction is to utilize the amino acid properties including hydrophobicity^27^, hydrophilicity^28^, surface accessibility^29^, flexibility^30^ and antigenicity^31^. In addition, there are methods using machine learning algorithms such as Hidden Markov Model (HMM)^32^, Artificial Neural Network (ANN)^33^ and Support Vector Machine (SVM)^34^ to locate antigenic epitopes. However, sequence length to normalize the epitope density never has been considered to measure the antigenic potential of a protein sequence at a genome wide level.

In the present study, we predicted ES proteins encoded in the *T. solium* genome, followed by functional annotation. Predicted ES proteins were functionally annotated in terms of similarity to other known proteins, biochemical pathways, gene ontologies, protein families and domains. ES proteins were also analyzed for number of antigenic regions using three different bioinformatics algorithms and searched for structural homologues using fold recognition algorithms. We developed a novel genomic measurement to evaluate the potential antigenicity of a secretome using the sequence length and the number of antigenic regions of ES proteins. This measurement was formalized as the Abundance of Antigenic Region (AAR) value. We also determine the AAR value for a set of 46 experimentally determined antigenic proteins of *T. solium* and for previously reported ES proteins of 12 parasitic helminth species. We believe that our genome wide exploration of ES proteins is a valuable resource for future experimental studies of the *T. solium* secretome. Our work represents a starting point to the characterization of the parasite secretome and it would contribute to a better comprehension of the host-parasite interactions.

## Results

### Prediction of Excretory/Secretory (ES) proteins of *T. solium* genome

**Figure 1:**
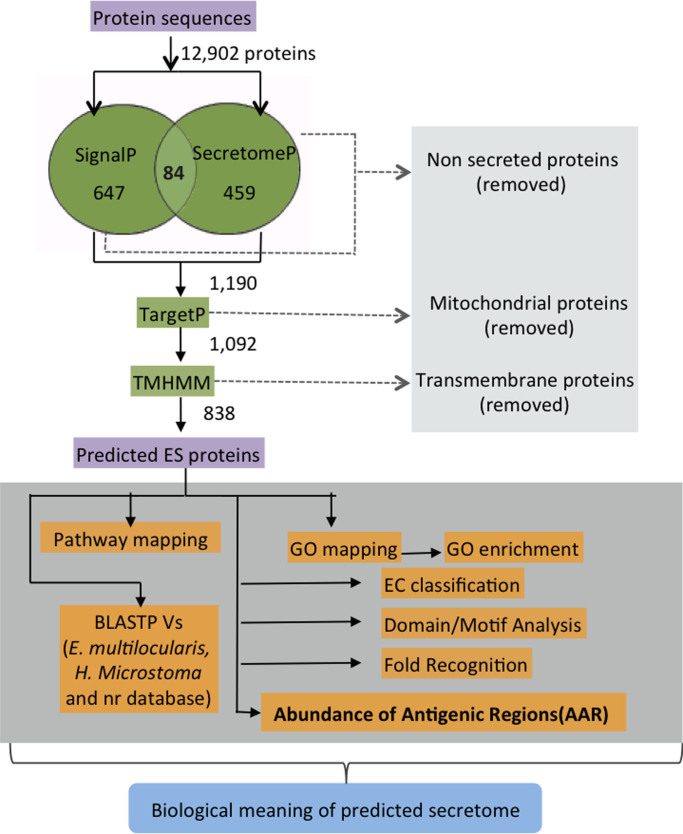
Bioinformatics pipeline to identify and annotate the ES proteins in *T. solium* genome.

The bioinformatics pipeline is summarized in Figure 1. Of the 12,902 proteins encoded in the *T. solium* genome^26^, we could annotate a total of 731 proteins as classical secretory proteins by SignalP^35^ and 543 proteins as non-classical secretory proteins by SecretomeP^36^. The classical and non-classical secretory proteins were merged, yielding a set of only 1190 different proteins because 84 proteins were shared between both predictions (see Venn diagram in Figure 1). The 1190 proteins were subsequently analyzed by TargetP^37^ to identify mitochondrial proteins. After that, 98 proteins were predicted as mitochondrial and were removed from the original set of 1190 proteins. The remaining 1092 proteins were scanned using TMHMM^38^ and for 254 proteins transmembrane regions were predicted. These transmembrane proteins were removed from the protein dataset. Finally, a total of 838 sequences were predicted as ES proteins by our bioinformatics pipeline (Figure 1). The 838 ES proteins represent the 6.5% of the total sequences of *T. solium* genome. The ES proteins were searched against the RNAseq and ESTs libraries from *T. solium* to analyze the percentage of ES proteins that are supported at RNA level. The access to its RNA data was kindly provided by *T. solium* consortium (unpublished data). Interestingly, we found RNA support for 347 ES proteins, representing 41.4% of the total *T. solium* secretome.

### Functional annotation of *T. solium* secretome

#### ES protein identification

Of the 838 ES proteins, 654 (81.6%) proteins show significant BLASTP matches with proteins deposited in the non-redundant (nr) database and 63 (7.5%) proteins represented significant BLASTP matches with hypothetical protein homologs. According to the sequence description of protein homologs, several ES proteins were indentified as diagnostic antigen gp50 (14 proteins), cystein-rich secretory protein (9 proteins), chorion class high cystein protein (6 proteins), oncosphere antigen a (5 proteins) and others.

#### Gene Ontology analysis

**Figure 2:**
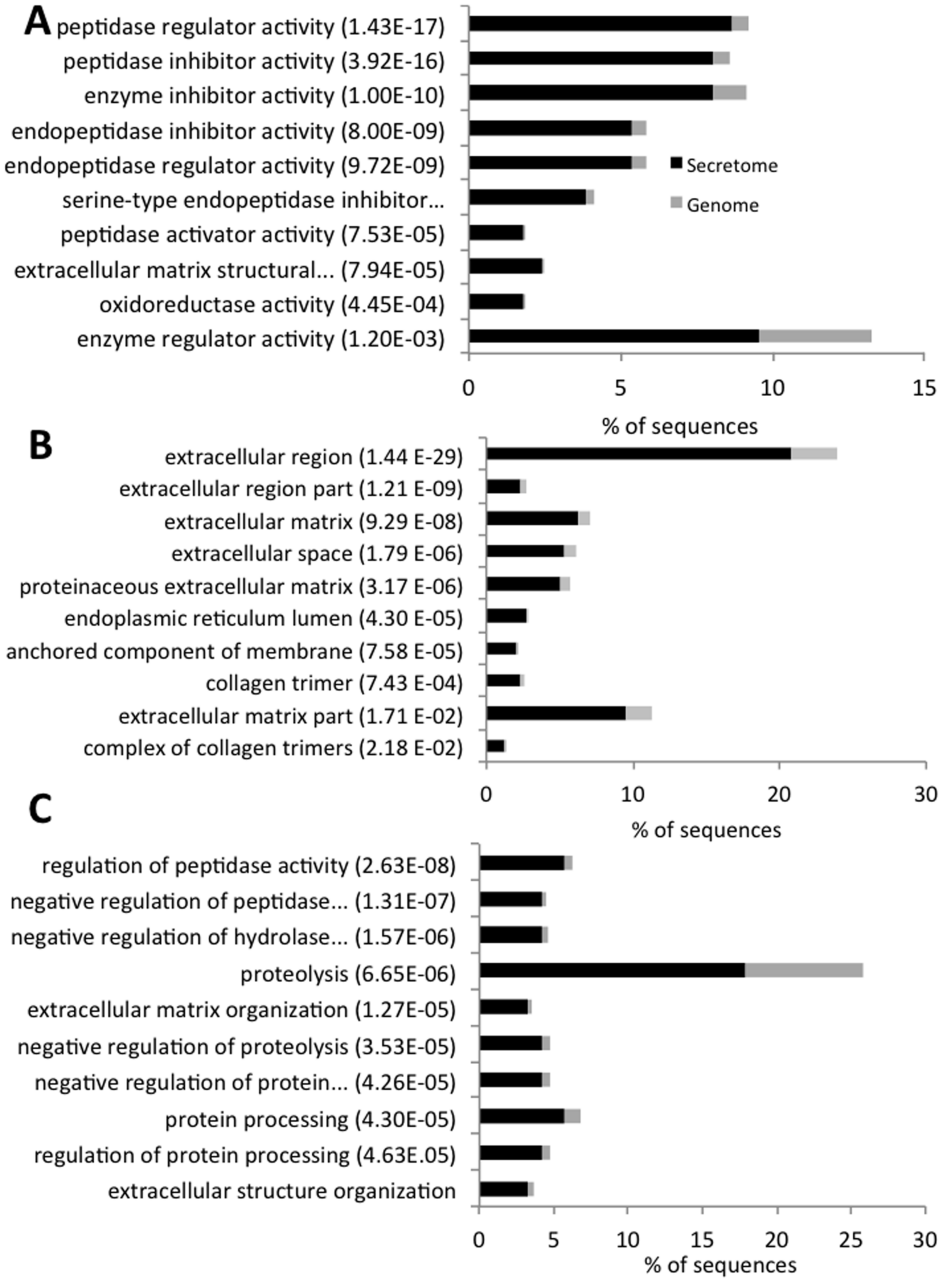
Gene Ontology distribution of ES proteins and non-ES proteins from *T. solium*. Distribution of Gene Ontology terms at level 2 for: (A) Molecular Function, (B) Cellular Component and (C) Biological Process.

ES proteins were annotated for Biological Process, Molecular Function and Cellular Components with Gene Ontology (GO) terms. Out of 838 ES proteins, 349 (41.6%) proteins were annotated with GO terms using Blast2GO^39^,^40^. In an effort to obtain more sequences with annotations, the 488 unannotated proteins were subjected to GO terms annotation using Argot2^41^. The advantage of Argot2 is that it exploits HMMER searches in addition to the typical BLAST searches and combines the clustering of GO terms based on their semantic similarities with a weighting scheme to annotate the query sequences^41^. After the analysis using Argot2, we can annotate 276 proteins from the 488 originally unannotated by Blast2GO^39^,^40^. In summary, of the 838 ES proteins, 625 (74.6%) proteins were annotated with 1429 different GO terms (835 for Biological Process, 231 for Cellular Component and 363 for Molecular Function) using the two annotation bioinformatics programs. The 12,064 non-ES proteins of the *T. solium* genome were also analyzed for GO terms annotation. After that, a total of 10,218 (84.7%) proteins were mapped with GO terms. The GO terms distribution to a second level category is provided in Figure 2 for ES and non-ES proteins from *T. solium* genome.

The most represented GO terms in the 838 ES proteins at Molecular Function category (Figure 2A) were: binding (42%) and catalytic activity (37%). The molecular function regulator and catalytic activity terms show an overrepresentation of annotated sequences in the ES proteins as compared to the distribution of the same terms for the non-ES proteins of *Taenia solium* genome (Figure 2A). Contrary, transporter activity and binding terms show a subrepresentation of annotated sequences in the secretome as compared to the distribution of the same terms for the non-ES proteins. The binding term predominantly includes at the third level subcategory the ion binding (13%), protein binding (11%), organic cyclic compound binding (11%), heterocyclic compound binding (11%) and small molecule binding (10%) terms. The catalytic activity term predominantly includes at the third level subcategory the hydrolase activity (10%), transferase activity (10%), oxidoreductase activity (3%), isomerase activity (1%), ligase activity (0.5%) and lyase activity (0.5%) terms.

The most represented GO terms in the ES proteins at Cellular Component category (Figure 2B) were: cell (28%), organelle (21%), membrane (21%), macromolecular complex (10%), extracellular region (9%) and membrane enclosed lumen (4%) terms. The extracellular matrix, extracellular region and membrane terms show an overrepresentation in the secretome as compared to the distribution of the same terms for the non-ES proteins (Figure 2B). The most represented GO terms in the 838 ES proteins at Biological Process category (Figure 2C) were: cellular process (18%), metabolic process (16%), single-organism process (14%), biological regulation (10%), response to stimulus (7%) and multicellular organism process (5%) terms. The biological adhesion, biological regulation and metabolic process terms show an overrepresentation in the secretome as compared with the distribution of the same terms for the non-ES proteins of *Taenia solium* genome.

#### Gene Ontology terms enrichment

**Figure 3:**
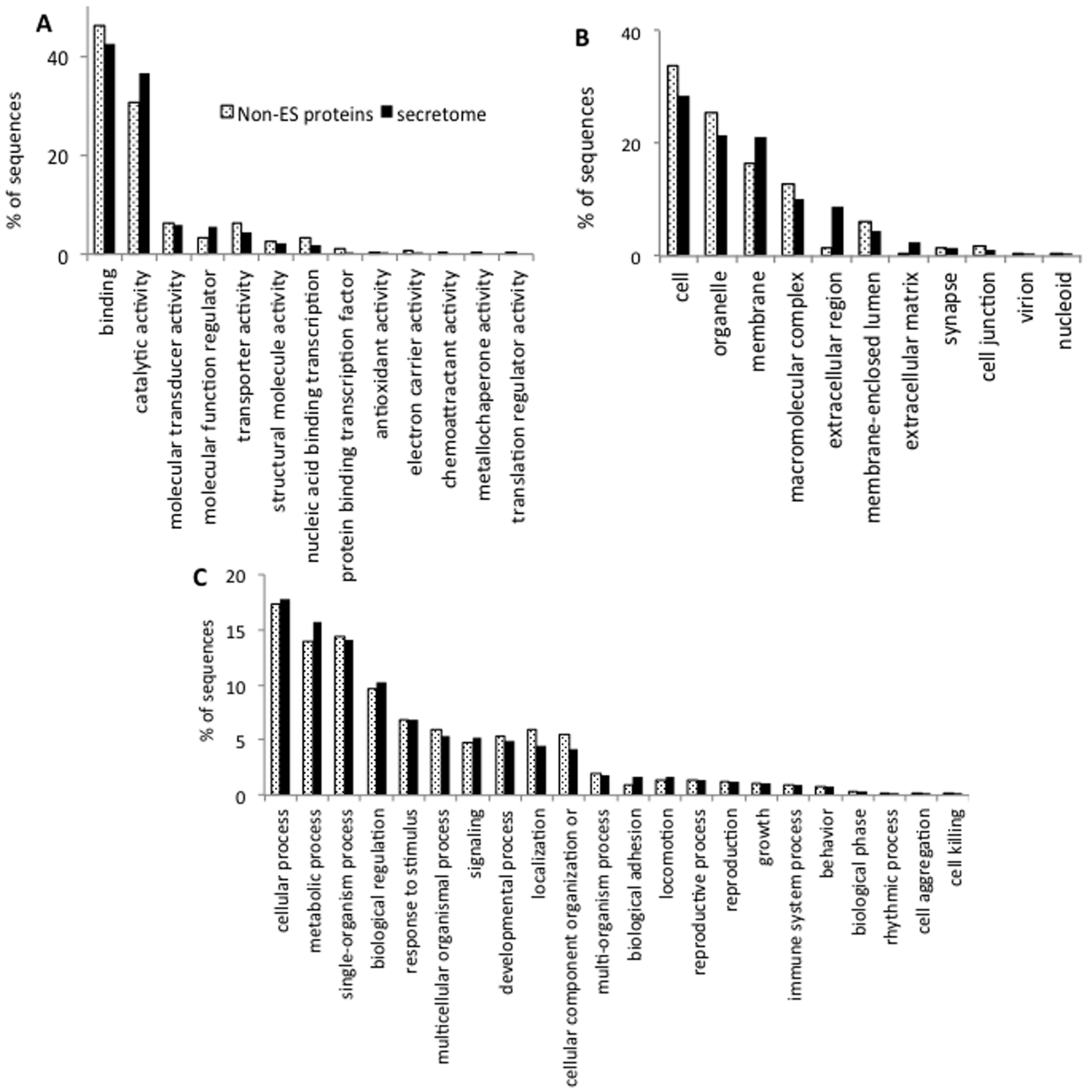
Gene Ontology enrichment of ES proteins as compared to the total proteins from *T. solium* genome. Significantly enrichments of Gene Ontology terms for: (A) Molecular Function, (B) Cellular Component and (C) Biological Process.

We analyze whether any GO term shows a significant enrichment in the secretome as compared to the expected by GO term distributions for all *T. solium* genome (Figure 3). In the molecular Function category a significant enrichment with terms related to the regulation of peptidase activities, extracellular matrix structural constituent and oxidoreductase activity was found (Figure 3A). The terms related to extracellular components, endoplasmic reticulum lumen and components anchored to membrane shows a significant enrichment in the Cellular Component category (Figure 3B). The terms that show a significant enrichment in Biological Process category were related to regulation of peptidase and hydrolase activity, proteolysis and extracellular structure organization (Figure 3C). The complete lists of significantly GO enrichments assigned to ES proteins are provided in Supplementary Tables S1–S3.

#### Pathway mapping

We used KAAS^42^,^43^,^44^ to annotate ES proteins to biochemical pathways. A total of 384 (45.8%) ES proteins were associated to 166 KEGG pathways. The most represented KEGG pathways are shown in Table 1 and full annotations are available in Supplementary Table S4. The two most frequently mapped KEGG pathways were protein processing in endoplasmic reticulum and Lysosome. Interestingly, four proteins were predicted as involved in antigen processing and presentation (ranking 23) which might play critical roles in host-parasite interactions.

#### Enzyme Code Distribution

**Figure 4:**
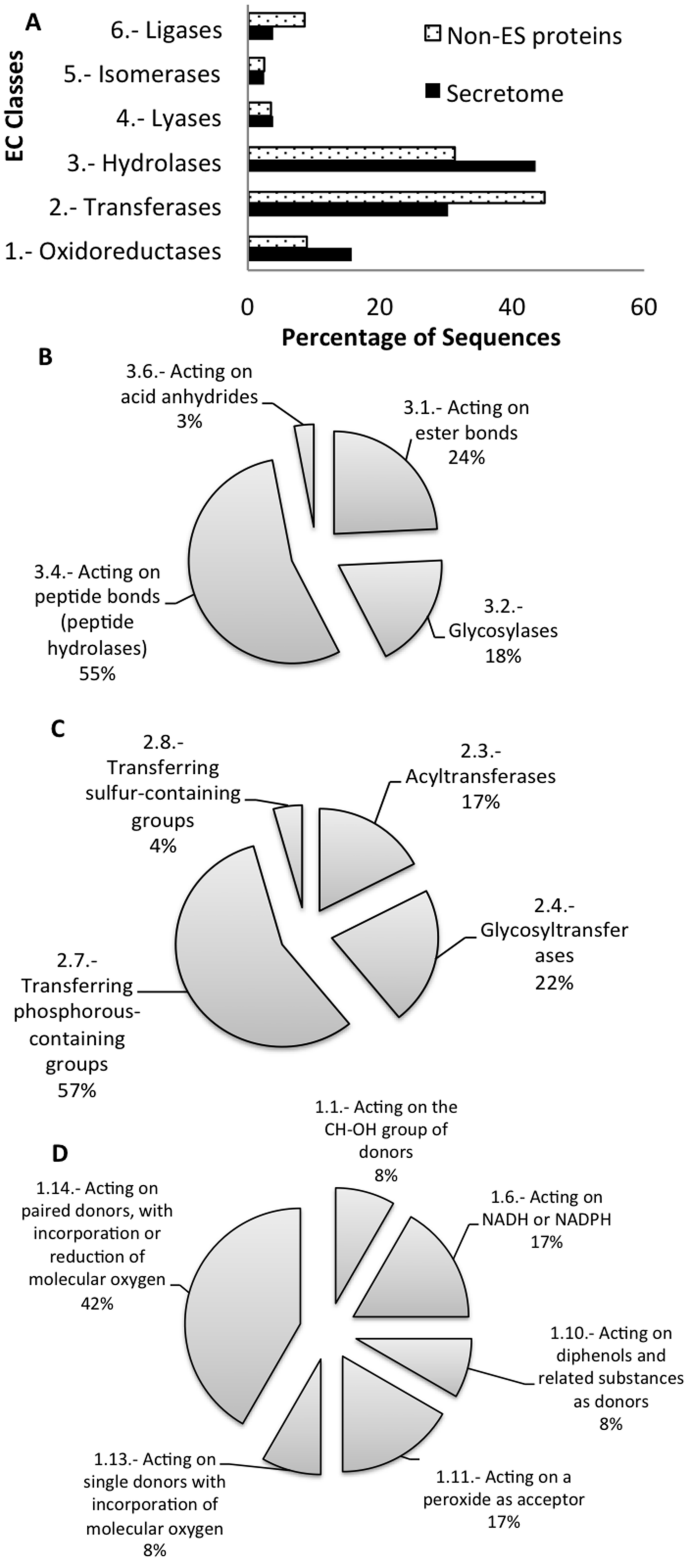
Enzyme commission classes and subclasses distribution of *T. solium* ES proteins. (A) EC classes for ES and non-ES proteins, (B) EC hydrolase subclasses for ES proteins, (C) EC transferase subclasses for ES proteins and (D) oxidoreductase subclasses for ES proteins.

We classified the enzymes contained in the ES proteins and in the non-ES proteins according to the six enzymes commission classes (Figure 4). The results show an overrepresentation of hydrolases, oxidoreductases and ligases in the ES proteins as compared to the same enzyme types for the non-ES proteins of *Taenia solium* genome (Figure 4A). The hydrolases represented 43% of the enzymes in the ES proteins, while this enzyme type represented 31% of the non-ES proteins (Figure 4A). The oxidoreductases represented 16% of the enzymes in the ES proteins, while this enzyme type only represented 9% of the non-ES proteins (Figure 4A). The three most represented EC Subclasses of Hydrolase enzymes were: acting on peptide bonds (peptide hydrolases) (18 proteins), acting on ester bonds (8) and glycosylases (6) (Figure 4B). The three most represented EC subclasses of Transferase enzymes were: transferring phosphorous-containing groups (13 proteins), glycosyltransferases (5) and acyltransferases (4) (Figure 4C). Finally, the most represented EC subclasses of oxidoreductases enzymes are shown in Figure 4D.

#### Analysis of protein domains and motifs

The annotation of ES proteins using InterProScan^45^,^46^ resulted in 491 protein families and domains. The most represented InterPro domains are shown in Table 2. The three most represented protein domains were the Immunoglobulin-like fold, CAP domain and fibronectin type III. Interestingly, the Immunoglobulin-like domains are involved in a variety of functions, including cell-cell recognition, cell-surface receptors, muscle structure and the immune system. The Taeniidae antigen was also overrepresented (ranking 14).

### Functional analyses of the specific *T. solium* secretome

We compared the 838 ES proteins against the genomes of *E. multilocularis* (Family: Taeniidae) and *H. microstome* (Family: Hymenolepididae) to discard the ES proteins with homologues in both genomes. These two species are the closest evolutionary related genomes to the *T. solium* genome that are sequenced to date^26^. From these analyses, we retrieved 121 ES proteins without homologues in both genomes (threshold e-value of 1 E^−3^). These 121 ES proteins also were BLASTed against all the non-redundant (nr) proteins of NCBI and we did not find any related protein homologue (threshold e-value of 1 E^−3^). Thus, these 121 proteins constitute the specific secretome of the *T. solium* genome and can be used as specific targets for *T. solium* infections. After mapping the set of 121 ES proteins to the InterPro and KEGG databases, we did not obtain protein sequences with functional annotations. Nonetheless, we annotated 39 sequences with 83 different GO terms using Argot2. However, the GO term enrichment analysis of these 39 sequences does not show statistically significant results as compared with GO distributions for all genome of *Taenia solium*. In an effort to obtain more functional information for this set of ES proteins, we subjected the 121 sequences to a fold recognition analysis using the Phyre2 algorithm^47^. Phyre2 algorithm was recently used as an alternative approach for functional annotation of novel protein sequences. In this regard, if the predicted structure for query protein is confident, the template protein functions can be tentatively assigned to the query protein. The confidence score of Phyre2 was established to 55% as the minimum cut-off value and the proteins with confidence scores equal to or higher than this cut-off value are shown in Table 3. The protein 08062.0.1 has a high structural similarity with the UPLC1 protein. Interestingly, the UPLC1 protein is an important regulator in cancer cell migration/invasion and in actin-based cytoskeletal remodeling^48^.

### The Abundance of Antigenic Regions (AAR) value

To evaluate the antigenicity potential of *T. solium* secretome the number of antigenic regions for each protein sequence was obtained using three different bioinformatics algorithms: the method reported by Kolaskar and Tongaonkar^31^, CBTOPE^34^ and BepiPred^32^. The Kolaskar^31^ method is a classical approach that uses the antigenicity propensity and physicochemical properties of amino acids to make the prediction of antigenic regions. The BepiPred^32^ method combine the hydrophilicity property of amino acids with a Hidden Markov Model (HMM) to predict B-cell epitopes. The CBTOPE^34^ method predicts conformational B-cell epitopes using the amino acid composition as an input feature for a Support Vector Machine (SVM) model. However, to normalize the number of antigenic regions by sequence length we introduce the Abundance of Antigenic Regions (AAR) value (see materials and methods). This normalization was applied to the results of the three bioinformatics methods used for antigenic prediction. The AAR value was used to define the number of amino acids between antigenic regions per sequence. Hence, low AAR values means that protein has more antigenic regions (more epitope density). We determined the AAR value for the 838 ES proteins and we found in average one antigenic region each 26.2 amino acids using the Kolaskar method (Table 4), while the AAR values using the CBTOPE^34^ and Bepipred^32^ methods, were of 105.7 and 93.6 respectively (Table 4). The three different AAR values obtained for ES proteins are due to the different number of antigenic regions predicted by each method. However, the three methods used for the prediction of antigenic regions show a consistently AAR difference between the ES and non-ES proteins obtained in each method (Table 4). Hence, we use the obtained AAR values by Kolaskar^31^ method for comparisons between protein datasets.

The AAR value for the 347 ES proteins that are supported at RNA level was of 26.2 (Table 4). The AAR value for the set of 121 ES proteins that is specific of *T. solium* genome was of 28.9, while the non-ES proteins have average one antigenic region each 42.1 amino acids (Table 4). The AAR value for the 48 ES proteins supported at RNA level which are specific of the *T. solium* secretome was of 28.3. Interestingly, all ES proteins datasets had twofold more antigenic regions in comparison with the non-ES proteins of the *T. solium* genome (Table 4). Hence the epitope density in ES proteins is higher than for non-ES proteins. For the validation of biological significance of AAR values, we calculated this value for a dataset of experimentally derived ES proteins of *T. solium* compiled from literature (see materials and methods). This set contained 46 protein sequences that have been experimentally reported to be useful in the diagnostic of human teniosis or neurocysticercosis (Supplementary Table S5). Interestingly, the AAR value for this antigenic protein dataset was 21.8, which is close to the calculated value for the secretome (Table 4). In contrast, the non-ES proteins showed an AAR value of 42.1. Interestingly, 44 (95.6%) of the 46 diagnostic proteins were found in our secretome (Supplementary Table S5). Furthermore, we also found RNA support for these 44 proteins (Supplementary Table S5). To test whether our obtained AAR values are similar to other known secretomes, we selected the secretomes of 12 helminth species which were recently reported in the Helminth Secretome Database (HSD)^2^ and their AAR values were calculated. Table 5 contains the AAR values for the 12 helminth secretomes (4 nematodes, 4 trematodes and 4 cestodes). Interestingly, the obtained AAR values for known helminth secretomes were very similar to that obtained for the *T. solium* secretome which is reported in this study (Table 5).

## Discussion

The cysticercosis is a neglected zoonotic infection caused by *T. solium* parasite. It is one of the WHO's lists of most neglected tropical diseases and the most prevalent human tapeworm. We have applied different bioinformatics approaches to identify and annotate all the predicted ES proteins encoded in the *T. solium* genome. To the best of our knowledge, the present study is the most comprehensive *in silico* collection of the *T. solium* secretome and it represented the 6.5% of the total proteins encoded in their genome. This proportion of ES proteins is in agreement with secretomes previously reported for other species^2^,^26^. The ES proteins can circulate in the extracellular space of an organism making them attractive as targets for novel therapeutics, because they may be more accessible to drugs than other proteins. Our *T. solium* secretome provides a rich source of potential drug targets, vaccine candidates or diagnostic proteins for developing new treatment and diagnostics strategies. In addition, our study contributes to increase the knowledge of the molecular mechanisms of host-parasite interaction. As well as to identify novel proteins with immunomodulatory properties that could be used as targets to control inflammatory processes of non-infectious diseases.

Functional information of the *T. solium* secretome was obtained through the analysis of Gene Ontology (GO) annotations of the 838 ES proteins. The top 10 GO term enrichment showed a statistical overrepresentation in the ES proteins of biological activities that are strongly related to the typical functions of secreted proteins (Figure 3). The GO terms related to extracellular matrix, endoplasmic reticulum lumen and anchored to membrane showed a significant enrichment in the Cellular Component category. The secretome of an organism includes all proteins secreted by the cell including those of the extracellular matrix, proteins shed from the cell membrane and vesicle proteins like microsomal vesicles^1^,^49^,^50^. The GO term enrichment related to the endoplasmic reticulum lumen suggests that, even with a correctly predicted signal peptide, some proteins can be resident of the endoplasmic reticulum. The top 10 GO term enrichment of Biological Process and Molecular Function showed a statistical overrepresentation in the ES proteins of peptidase activities, extracellular organization and cell adhesion terms. Proteins with peptidase domains have been previously reported to be involved in virulence activity in several helminth species^51^. Several ES proteins were predicted to be involved in antigen processing and presentation pathway. Interestingly, there is evidence that secreted glycoantigens by cysticerci can modulate the host inflammatory response through the activation of dendritic cells in the experimental murine cysticercosis caused by *T. crassiceps*^52^. However, the relevance of ES proteins on the modulation of host-parasite relationships has not been studied in human cysticercosis. Although, it is well known that helminth ES proteins can modulate the host immune system during the infection for parasite survival^13^,^14^,^15^.

The functional annotations found in the *T. solium* secretome by GO term enrichment, pathway mapping, enzyme code distribution and protein domain analysis strengthened our bioinformatics workflow to be useful to predict secretomes in other genomes. However, it is clear that integration of bioinformatics strategies with RNAseq data can improve the identification of expressed secretomes. Interestingly, the 41.4% of our secretome was supported at RNA level (unpublished data). The 121 ES proteins specific of *T. solium* secretome represents potential novel drug or vaccine targets for therapeutic strategies and denotes the importance of future experimental research to characterize this protein dataset. The proteins of this dataset are not shared with other sequenced organisms, suggesting that it can be explored as diagnostic proteins for specific *T. solium* infections. The *T. solium* is unable to synthesize the amino-acid lysine and among the secreted proteins we found enzymes able to degrade lysine-containing peptides. This finding is an example of the complex host-parasite interactions. The presence of lytic proteins in our secretome, suggest that these proteins can be used to cut down nutrients making them more accessible for the parasite or to cut down immune response-related molecules that could induce parasite damage^53^,^54^,^55^,^56^,^57^,^58^. Interestingly, the hydrolases and oxidoreductases showed an overrepresentation in the secretome as compared to the distribution of the same terms for the non-ES proteins of *Taenia solium* genome. It is in agreement with the considerable enrichment of this enzyme types found in other experimentally determined secretomes^50^,^59^,^60^.

Previously was suggested that high epitope density in a single protein molecule significantly enhances their antigenicity and immunogenicity^61^. Here, we found that experimental determined antigenic proteins have more antigenic density, measured by the normalization of the number of antigenic regions by sequence length (AAR values in Tables 4 and 5). It is, in fact, a manageable metric which reflects the epitope density of a protein. To our knowledge, AAR is the first example of a tool implementing antigenic regions and sequence length to estimate the antigenicity of a protein at genome wide level. Nearly 40% of predicted ES proteins remain unannotated in the Helminth Secretome Database (HSD)^2^. The sequence annotation results obtained for the *T. solium* specific secretome, which were based in BLAST and HMMER searches, fold recognition strategies and AAR values, suggest that these strategies can be used to enhance the annotations of known secretomes. The Abundance of Antigenic Regions (AAR) value for the *T. solium* secretome (Table 4) showed that these proteins are enriched of antigenic regions as compared to the non-ES proteins. Interestingly, the AAR values for the ES proteins were very similar to that obtained for the diagnostic proteins, suggesting their potential use in the diagnosis of *T. solium* infections (Table 4). In addition, the obtained AAR values for known helminth secretomes were very similar to that obtained for *T. solium* secretome (Table 5). These results demonstrated the utility of the AAR value as a novel genomic measurement to evaluate the potential antigenicity of ES proteins at genome wide level. The traditional cloning of the proteins for immunization purposes is clearly not feasible on a genomic scale. The AAR approach is cost effective and can guide a genome wide search for antigenic proteins of therapeutic, diagnosis and immunological interest.

The use of different algorithms to make the prediction of antigenic regions could potentially improve the predictions. In this work, we obtained the AAR values using the number of antigenic regions predicted from three independent algorithms, the CBTOPE^34^ which is based in a Support Vector Machine (SVM) model, the BepiPred^32^ which is based in a Hidden Markov Model (HMM) and Kolaskar^31^ that uses the antigenicity propensity and physicochemical properties of amino acids to make the prediction of antigenic regions. Although, the obtained AAR values using Kolaskar^31^ method shows more antigenic regions per protein than the AAR values obtained using CBTOPE^34^ and BepiPred^32^, there is a consistently difference of AAR values between ES and non-ES proteins for each method (Tables 4–5).

The *T. solium* ES proteins could be used as antigens to capture antibodies from infected patients. Subsequently, the antibodies can be used to directly detect the ES antigens in infected patients through a sandwich ELISA. Actually, the human NC diagnosis has not high sensibility and specificity to establish the definitive NC diagnosis in patients with neurological diseases. The HP10 monoclonal antibody is one of the best proteins used for immunodiagnosis. However, the HP10 is only effective for the detection and the follow-up of the most severe forms of NC (this is when vesicular cysticercis are located in subarachnoid space at the base^62^). Although, novel ES antigens from oncosphere stage has been recently suggested for NC diagnosis^25^,^63^. However, the immunoassays in pigs using *T. solium* ES or total antigens have been demonstrated a low sensibility and many false positives and false negatives^64^. The experimental study of the ES proteins identified in this work will confirm the proteins that can be candidate for use in the development of new diagnostic tests and new disease treatments. However, protein functions are strongly context-dependent and further experimental analyses are needed to improve the reliability of the functional interpretation of our results. Additionally, further studies on the proteomic level are highly desirable to confirm the predicted secretome reported herein.

## Methods

### Prediction of Excretory/Secretory (ES) proteins of *T. solium* genome

The bioinformatics pipeline is summarized in Figure 1. We started out with 12,902 protein sequences of the *T. solium* genome^26^. For all of these proteins the SignalP (version 4.1)^35^ and SecretomeP (version 2.0)^36^ algorithms were applied. SignalP was used to predict classically secreted proteins, setting the option for eukaryote organisms and the positional limit of 70 residues for truncation before submitting it to the neural networks algorithm. The input sequences also may include TM regions and the D-cutoff values were setting as default. SecretomeP was used to predict the non-classical secreted proteins using the default options for mammalian organisms. All the classical and non-classical secretory proteins were merged together and the resulting list was scanned by TargetP^37^ to predict the mitochondrial proteins, using at 95% of specificity and the default options for non-plant organisms. The mitochondrial proteins predicted by TargetP were discarded from the protein data set. The resulting ES proteins were subsequently scanned for the presence of transmembrane helices by TMHMM (version 2.0)^38^ and protein sequences exhibiting transmembrane helices were also excluded from the final protein data set.

### Functional annotation and comparative analysis of ES proteins

The ES proteins were functionally annotated using several bioinformatics tools. For identifying homologous proteins, ES proteins were BLASTed (BLASTP) against the non-redundant (nr) database using the Blast2GO package. The E-value cut-off was set at 1.0 E^−3^. Supported by Blast2GO^39^,^40^,^65^,^66^ ES proteins were functionally mapped to GO terms and annotated by setting the following parameters: E-Value-Hit-Filter: 1.0 E^−3^; Annotation cut-off: 55; GO weight: 5; Hsp-Hit Coverage cut-off: 0.

The ES proteins were also mapped to Gene Ontology terms using Argot2^41^ by setting the Total Score (TS) to ≥200. Additionally, ES proteins were associated to protein families and domains through InterProScan^45^,^46^. Blast2GO was used to identify the statistically enriched GO terms represented in the ES proteins setting the term filter value to 0.05 and the term filter mode to FDR. The KAAS was used for mapping ES proteins to KEGG pathways and to KEGG BRITE objects using the BBH (bi-directional best hit) method to assign the orthologs and the representative genes data set was setting for eukaryotes^42^,^43^,^44^.

### Functional analyses of the specific *T. solium* secretome

The 838 ES proteins were searched for sequence similarity against the *Hymenolepis microstoma* (Family: Hymenolepididae) and *E. multilocularis* (Family: Taeniidae) genomes^26^ using BLASTP (E-value cut-off was set at 1.0 E^−3^) to obtain the specific secretome of *T. solium*. The number of antigenic regions was calculated using the methods Kolaskar and Tongoankar^31^, CBTOPE^34^ and BepiPred^32^ for each protein. The Abundance of Antigenic Regions (AAR) was calculated as follows for each method:
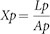


*Xp*: The relative abundance of antigenic regions in protein *p*

*Lp*: The sequence length in protein *p*

*Ap*: The number of antigenic regions in protein *p*

The AAR value was introduced to define the number of amino acids between antigenic regions for each protein. This value was scored as the ratio between the sequence lengths to the number of predicted antigenic regions for each protein. Hence, the final value determines the number of amino acids that are needed to find one antigenic region in the corresponding sequence. The dataset of experimentally-determined proteins used to diagnose human *T. solium* infections was compiled from a search at NCBI database. After that, we found 46 different proteins, at the sequence level, that have been experimentally reported to be useful for the diagnostic of human teniosis or neurocysticercosis (Supplementary Table S5). The ES protein sequences also were submitted to Phyre2 program^47^ using the default options and the twenty top scoring matches (if any) were retained for each protein. The Phyre 2 result is based in secondary structure prediction coupled to fold-recognition and three-dimensional structure predictions^47^.

## Author Contributions

S.G., L.A.P., H.P.F., V.A.C.R. and A.O.L. generated and conducted the bioinformatics analyses. S.G. and A.O.L. performed the statistical analysis. L.A.P. and A.O.L. wrote the manuscript. X.S., E.S., G.F., R.J.B., J.P.L. and L.P.Y. examined the data. A.O.L. conceived the project, generated data, conducted bioinformatics analyses and coordinated the draft manuscript. All authors edited and approved the final manuscript.

## Additional information

**How to cite this article**: Gomez, S. *et al*. Genome analysis of Excretory/Secretory proteins in *Taenia solium* reveals their Abundance of Antigenic Regions (AAR). *Sci. Rep.* 5, 9683; DOI:10.1038/srep09683 (2015).

## Supplementary Material

Supplementary Information Supplementary Information

## Figures and Tables

**Table 1:** Top 15 most represented KEGG pathways in *T. solium* secretome

Pathway name	Number of the represented ES proteins (%)
Protein processing in endoplasmic reticulum	11 (1.31)
Lysosome	10 (1.19)
Pathways in cancer	10 (1.19)
Focal adhesion	9 (1.07)
Hippo signaling pathway	7 (0.84)
Proteoglycans in cancer	7 (0.84)
Purine metabolism	5 (0.60)
Wnt signaling pathway	5 (0.60)
PI3K-Akt signaling pathway	5 (0.60)
Phagosome	5 (0.60)
Protein digestion and absorption	5 (0.60)
Alcoholism	5 (0.60)
Epstein-Barr virus infection	5 (0.60)
Glycerophospholipid metabolism	4 (0.48)
Pyrimidine metabolism	4 (0.48)

**Table 2:** Top 15 most represented protein domains in *T. solium* secretome

InterPro code	InterPro description	Number of ES proteins (%)
IPR013783	Immunoglobulin-like fold	32 (3.81)
IPR014044	CAP domain	18 (2.14)
IPR003961	Fibronectin, type III	17 (2.02)
IPR007110	Immunoglobulin-like	16 (1.90)
IPR001283	Allergen V5/Tpx-1-related	15 (1.78)
IPR002223	Proteinase inhibitor I2, Kunitz metazoa	14 (1.67)
IPR020901	Proteinase inhibitor I2, Kunitz, conserved site	12 (1.43)
IPR003599	Immunoglobulin subtype	9 (1.07)
IPR011009	Protein kinase-like domain	9 (1.07)
IPR013083	Zinc finger, RING/FYVE/PHD-type	9 (1.07)
IPR002126	Cadherin	8 (0.95)
IPR015919	Cadherin-like	8 (0.95)
IPR000719	Protein kinase, catalytic domain	8 (0.95)
IPR008860	Taeniidae antigen	8 (0.95)
IPR007087	Zinc finger, C2H2	8 (0.95)

**Table 3:** Phyre2 confident predictions found in the *T. solium* specific secretome

Gene ID	Top structural hit	Confidence (%)	Sequence identity (%)	Template information
08062.0.1	2b0b	86.7	18	PDB header: metal binding protein; Chain: F: PDB Molecule:uplc1
47522.0.1	2dcw	78.5	100	PDB header: antimicrobial protein; Chain: A: PDB Molecule:tachystatin-b2
10029.7.1	1yfo	69.0	47	(Phosphotyrosine protein) phosphatases II; Higher-molecular-weight phosphotyrosine protein phosphatases
43027.1	2fd5	59.9	42	Tetracyclin repressor-like, C-terminal domain
69637.1	1xak	58.7	47	Immunoglobulin-like beta-sandwich; Accessory protein X4 (ORF8, ORF7a)

**Table 4:** Abundance of Antigenic Regions (AAR) for different *T. solium* protein datasets

Protein dataset	Number of proteins in the dataset	Average of AAR values (Kolaskar)	Average of AAR values (CBTOPE)	Average of AAR values (BepiPred)
Secretome	838	26.2	105.7	93.6
Secretome supported at RNA level	347	26.2	108.2	101.9
Specific secretome	121	28.9	85.4	76.7
Specific secretome supported at RNA level	48	28.3	84.4	83.5
Experimentally determined ES proteins	46	21.7	74.3	81.3
Non-ES proteins from *T. solium* genome	12064	42.1	126.5	102.1

**Table 5:** Abundance of Antigenic Regions (AAR) for different known helminth secretomes

ES proteins	Relative Density of Antigenic Regions (Kolaskar)	Average of AAR values (CBTOPE)	Average of AAR values (BepiPred)
**Nematodes**			
*Haterorhabditis bacteriophora*	26.4	96.5	105.0
*Caenorhabditis brenneri*	26.9	102.1	96.0
*Caenorhabditis japonica*	26.7	97.8	94.8
*Heterodera glycines*	29.1	100.6	97.7
**Trematodes**			
*Echinostoma paraensei*	24.6	78.9	82.4
*Fasciola gigantica*	28.2	82.0	80.5
*Opisthorchis viverrini*	26.6	86.6	73.6
*Paragonimus westermani*	26.3	68.1	77.8
**Cestodes**			
*Echinococcus multilocularis*	28.0	91.0	92.0
*Mesocestoides corti*	26.6	84.8	65.9
*Moniezia expansa*	27.3	95.5	95.0
*Spirometra erinaceieuropaei*	27.6	111.6	78.9
***Taenia solium***	26.2	105.7	93.6

## References

[b1] Tjalsma H., Bolhuis A., Jongbloed J. D., Bron S. & van Dijl J. M. Signal peptide-dependent protein transport in *Bacillus subtilis*: a genome-based survey of the secretome. Microbiol Mol Biol Rev 64, 515–547 (2000). 10974125 10.1128/mmbr.64.3.515-547.2000 PMC99003

[b2] Garg G. & Ranganathan S. Helminth secretome database (HSD): a collection of helminth excretory/secretory proteins predicted from expressed sequence tags (ESTs). BMC Genomics 13 **Suppl 7** S8 (2012). 23281827 10.1186/1471-2164-13-S7-S8 PMC3546426

[b3] Kim S. H. *et al.* Structural and binding properties of two paralogous fatty acid binding proteins of *Taenia solium* metacestode. PLoS Negl Trop Dis 6, e1868 (2012). 23150743 10.1371/journal.pntd.0001868 PMC3493614

[b4] Bhattacharjee S., Stahelin R. V. & Haldar K. Host targeting of virulence determinants and phosphoinositides in blood stage malaria parasites. Trends Parasitol 28, 555–562 (2012). 23084821 10.1016/j.pt.2012.09.004 PMC3501602

[b5] Burgos-Portugal J. A., Kaakoush N. O., Raftery M. J. & Mitchell H. M. Pathogenic potential of *Campylobacter ureolyticus*. Infect Immun 80, 883–890 (2012). 22124656 10.1128/IAI.06031-11 PMC3264317

[b6] Soblik H. *et al.* Life cycle stage-resolved proteomic analysis of the excretome/secretome from *Strongyloides ratti*--identification of stage-specific proteases. Mol Cell Proteomics 10, M111 010157 (2012). 21964353 10.1074/mcp.M111.010157 PMC3237078

[b7] Garcia H. H., Moro P. L. & Schantz P. M. Zoonotic helminth infections of humans: echinococcosis, cysticercosis and fascioliasis. Curr Opin Infect Dis 20, 489–494 (2007). 17762782 10.1097/QCO.0b013e3282a95e39

[b8] de Aluja A. S. Cysticercosis in the pig. Curr Top Med Chem 8, 368–374 (2008). 18393899 10.2174/156802608783790794

[b9] Sciutto E. *et al.* *Taenia solium* disease in humans and pigs: an ancient parasitosis disease rooted in developing countries and emerging as a major health problem of global dimensions. Microbes Infect 2, 1875–1890 (2000). 11165932 10.1016/s1286-4579(00)01336-8

[b10] Garcia H. H., Gonzalez A. E. & Gilman R. H. Cysticercosis of the central nervous system: how should it be managed? Curr Opin Infect Dis 24, 423–427 (2011). 21788891 10.1097/QCO.0b013e32834a1b20 PMC3272367

[b11] Del Brutto O. H. Neurocysticercosis. Handb Clin Neurol 121, 1445–1459 (2014). 24365429 10.1016/B978-0-7020-4088-7.00097-3

[b12] Mewara A., Goyal K. & Sehgal R. Neurocysticercosis: A disease of neglect. Trop Parasitol 3, 106–113 (2013). 24470993 10.4103/2229-5070.122111 PMC3889086

[b13] Marcilla A. *et al.* Extracellular vesicles from parasitic helminths contain specific excretory/secretory proteins and are internalized in intestinal host cells. PLoS One 7, e45974 (2012). 23029346 10.1371/journal.pone.0045974 PMC3454434

[b14] Martinez-Ibeas A. M., Gonzalez-Lanza C. & Manga-Gonzalez M. Y. Proteomic analysis of the tegument and excretory-secretory products of *Dicrocoelium dendriticum* (Digenea) adult worms. Exp Parasitol 133, 411–420 (2013). 23357649 10.1016/j.exppara.2013.01.010

[b15] Hewitson J. P., Grainger J. R. & Maizels R. M. Helminth immunoregulation: the role of parasite secreted proteins in modulating host immunity. Mol Biochem Parasitol 167, 1–11 (2009). 19406170 10.1016/j.molbiopara.2009.04.008 PMC2706953

[b16] Bai X. *et al.* Inhibition of mammalian muscle differentiation by excretory secretory products of muscle larvae of *Trichinella spiralis* in vitro. Parasitol Res 110, 2481–2490 (2012). 22200963 10.1007/s00436-011-2789-2

[b17] Gonzalez-Miguel J. *et al.* Excretory/secretory antigens from *Dirofilaria immitis* adult worms interact with the host fibrinolytic system involving the vascular endothelium. Mol Biochem Parasitol 181, 134–140 (2012). 22050927 10.1016/j.molbiopara.2011.10.010

[b18] Baska P., Norbury L. J., Wisniewski M., Januszkiewicz K. & Wedrychowicz H. Excretory/secretory products of *Fasciola hepatica* but not recombinant phosphoglycerate kinase induce death of human hepatocyte cells. Acta Parasitol 58, 215–217 (2013). 23666658 10.2478/s11686-013-0126-x

[b19] Virginio V. G. *et al.* Excretory/secretory products from in vitro-cultured *Echinococcus granulosus* protoscoleces. Mol Biochem Parasitol 183, 15–22 (2012). 22261090 10.1016/j.molbiopara.2012.01.001

[b20] Rahman M. *et al.* Characterization of hydrophobic-ligand-binding proteins of *Taenia solium* that are expressed specifically in the adult stage. Parasitology 139, 1361–1374 (2012). 22657393 10.1017/S0031182012000613

[b21] Terrazas C. A. *et al.* Cestode antigens induce a tolerogenic-like phenotype and inhibit LPS inflammatory responses in human dendritic cells. Int J Biol Sci 7, 1391–1400 (2011). 22110390 10.7150/ijbs.7.1391 PMC3221946

[b22] Victor B. *et al.* Use of expressed sequence tags as an alternative approach for the identification of *Taenia solium* metacestode excretion/secretion proteins. BMC Res Notes 6, 224 (2013). 23742691 10.1186/1756-0500-6-224 PMC3686625

[b23] Tato P. *et al.* A cysteine protease from *Taenia solium* metacestodes induce apoptosis in human CD4+ T-cells. Parasitol Res 92, 197–204 (2004). 14652742 10.1007/s00436-003-1008-1

[b24] Molinari J. L. *et al.* *Taenia solium*: a cysteine protease secreted by metacestodes depletes human CD4 lymphocytes in vitro. Exp Parasitol 94, 133–142 (2000). 10831377 10.1006/expr.2000.4490

[b25] Zimic M. J. *et al.* Comparison of the peptidase activity in the oncosphere excretory/secretory products of *Taenia solium* and *Taenia saginata*. J Parasitol 93, 727–734 (2007). 17918349 10.1645/GE-959R.1

[b26] Tsai I. J. *et al.* The genomes of four tapeworm species reveal adaptations to parasitism. Nature 496, 57–63 (2013). 23485966 10.1038/nature12031 PMC3964345

[b27] Kyte J. & Doolittle R. F. A simple method for displaying the hydropathic character of a protein. J Mol Biol 157, 105–132 (1982). 7108955 10.1016/0022-2836(82)90515-0

[b28] Parker J. M., Guo D. & Hodges R. S. New hydrophilicity scale derived from high-performance liquid chromatography peptide retention data: correlation of predicted surface residues with antigenicity and X-ray-derived accessible sites. Biochemistry 25, 5425–5432 (1986). 2430611 10.1021/bi00367a013

[b29] Emini E. A., Hughes J. V., Perlow D. S. & Boger J. Induction of hepatitis A virus-neutralizing antibody by a virus-specific synthetic peptide. J Virol 55, 836–839 (1985). 2991600 10.1128/jvi.55.3.836-839.1985 PMC255070

[b30] Vihinen M., Torkkila E. & Riikonen P. Accuracy of protein flexibility predictions. Proteins 19, 141–149 (1994). 8090708 10.1002/prot.340190207

[b31] Kolaskar A. S. & Tongaonkar P. C. A semi-empirical method for prediction of antigenic determinants on protein antigens. FEBS Lett 276, 172–174 (1990). 1702393 10.1016/0014-5793(90)80535-q

[b32] Larsen J. E., Lund O. & Nielsen M. Improved method for predicting linear B-cell epitopes. Immunome Res 2, 2 (2006). 16635264 10.1186/1745-7580-2-2 PMC1479323

[b33] Saha S. & Raghava G. P. Prediction of continuous B-cell epitopes in an antigen using recurrent neural network. Proteins 65, 40–48 (2006). 16894596 10.1002/prot.21078

[b34] Ansari H. R. & Raghava G. P. Identification of conformational B-cell Epitopes in an antigen from its primary sequence. Immunome Res 6, 6 (2010). 20961417 10.1186/1745-7580-6-6 PMC2974664

[b35] Bendtsen J. D., Nielsen H., von Heijne G. & Brunak S. Improved prediction of signal peptides: SignalP 3.0. J Mol Biol 340, 783–795 (2004). 15223320 10.1016/j.jmb.2004.05.028

[b36] Bendtsen J. D., Jensen L. J., Blom N., Von Heijne G. & Brunak S. Feature-based prediction of non-classical and leaderless protein secretion. Protein Eng Des Sel 17, 349–356 (2004). 15115854 10.1093/protein/gzh037

[b37] Emanuelsson O., Nielsen H., Brunak S. & von Heijne G. Predicting subcellular localization of proteins based on their N-terminal amino acid sequence. J Mol Biol 300, 1005–1016 (2000). 10891285 10.1006/jmbi.2000.3903

[b38] Krogh A., Larsson B., von Heijne G. & Sonnhammer E. L. Predicting transmembrane protein topology with a hidden Markov model: application to complete genomes. J Mol Biol 305, 567–580 (2001). 11152613 10.1006/jmbi.2000.4315

[b39] Conesa A. & Gotz S. Blast2GO: A comprehensive suite for functional analysis in plant genomics. Int J Plant Genomics 2008, 619832 (2008). 18483572 10.1155/2008/619832 PMC2375974

[b40] Conesa A. *et al.* Blast2GO: a universal tool for annotation, visualization and analysis in functional genomics research. Bioinformatics 21, 3674–3676 (2005). 16081474 10.1093/bioinformatics/bti610

[b41] Falda M. *et al.* Argot2: a large scale function prediction tool relying on semantic similarity of weighted Gene Ontology terms. BMC Bioinformatics 13 **Suppl 4** S14 (2012). 22536960 10.1186/1471-2105-13-S4-S14 PMC3314586

[b42] Moriya Y., Itoh M., Okuda S., Yoshizawa A. C. & Kanehisa M. KAAS: an automatic genome annotation and pathway reconstruction server. Nucleic Acids Res 35, W182–185 (2007). 17526522 10.1093/nar/gkm321 PMC1933193

[b43] Kanehisa M. The KEGG database. Novartis Found Symp 247, 91–101; discussion 101–103, 119–128, 244–152 (2002). 12539951

[b44] Kanehisa M. *et al.* From genomics to chemical genomics: new developments in KEGG. Nucleic Acids Res 34, D354–357 (2006). 16381885 10.1093/nar/gkj102 PMC1347464

[b45] Zdobnov E. M. & Apweiler R. InterProScan--an integration platform for the signature-recognition methods in InterPro. Bioinformatics 17, 847–848 (2001). 11590104 10.1093/bioinformatics/17.9.847

[b46] Quevillon E. *et al.* InterProScan: protein domains identifier. Nucleic Acids Res 33, W116–120 (2005). 15980438 10.1093/nar/gki442 PMC1160203

[b47] Kelley L. A. & Sternberg M. J. Protein structure prediction on the Web: a case study using the Phyre server. Nat Protoc 4, 363–371 (2009). 19247286 10.1038/nprot.2009.2

[b48] Luo Y. *et al.* Loss of ASAP3 destabilizes cytoskeletal protein ACTG1 to suppress cancer cell migration. Mol Med Rep 9, 387–394 (2014). 24284654 10.3892/mmr.2013.1831

[b49] Garg G. & Ranganathan S. In silico secretome analysis approach for next generation sequencing transcriptomic data. BMC Genomics 12 **Suppl 3** S14 (2011). 22369360 10.1186/1471-2164-12-S3-S14 PMC3333173

[b50] Caccia D., Dugo M., Callari M. & Bongarzone I. Bioinformatics tools for secretome analysis. Biochim Biophys Acta 1834, 2442–2453 (2013). 23395702 10.1016/j.bbapap.2013.01.039

[b51] McVeigh P., Maule A. G., Dalton J. P. & Robinson M. W. *Fasciola hepatica* virulence-associated cysteine peptidases: a systems biology perspective. Microbes Infect 14, 301–310 (2012). 22178015 10.1016/j.micinf.2011.11.012

[b52] Terrazas C. A., Alcantara-Hernandez M., Bonifaz L., Terrazas L. I. & Satoskar A. R. Helminth-excreted/secreted products are recognized by multiple receptors on DCs to block the TLR response and bias Th2 polarization in a cRAF dependent pathway. Faseb J 27, 4547–4560 (2012). 23907435 10.1096/fj.13-228932 PMC3804751

[b53] Vargas-Parada L., Solis C. F. & Laclette J. P. Heat shock and stress response of *Taenia solium* and *T. crassiceps* (Cestoda). Parasitology 122, 583–588 (2001). 11393832 10.1017/s0031182001007764

[b54] Vargas-Parada L. & Laclette J. P. Role of the calcareous corpuscles in cestode physiology: a review. Rev Latinoam Microbiol 41, 303–307 (1999). 10932772

[b55] Laclette J. P. *et al.* Paramyosin inhibits complement C1. J Immunol 148, 124–128 (1992). 1727860

[b56] Baig S. *et al.* Purification and characterization of a metacestode cysteine proteinase from *Taenia solium* involved in the breakdown of human IgG. Parasitology 131, 411–416 (2005). 16178363 10.1017/s0031182005007821

[b57] Yan H. L. *et al.* Calcium-dependent proapoptotic effect of *Taenia solium* metacestodes annexin B1 on human eosinophils: a novel strategy to prevent host immune response. Int J Biochem Cell Biol 40, 2151–2163 (2008). 18378486 10.1016/j.biocel.2008.02.018

[b58] Gao Y. J., Yan H. L., Ding F. X., Lu Y. M. & Sun S. H. Annexin B1 at the host-parasite interface of the *Taenia solium* cysticercus: Secreted and associated with inflammatory reaction. Acta Trop 101, 192–199 (2007). 17349964 10.1016/j.actatropica.2006.10.014

[b59] Hori C. *et al.* Temporal alterations in the secretome of the selective ligninolytic fungus *Ceriporiopsis subvermispora* during growth on aspen wood reveal this organism's strategy for degrading lignocellulose. Appl Environ Microbiol 80, 2062–2070 (2014). 24441164 10.1128/AEM.03652-13 PMC3993130

[b60] Giddey K. *et al.* Comprehensive analysis of proteins secreted by *Trichophyton rubrum* and *Trichophyton violaceum* under in vitro conditions. J Proteome Res 6, 3081–3092 (2007). 17622167 10.1021/pr070153m

[b61] Liu W. & Chen Y. H. High epitope density in a single protein molecule significantly enhances antigenicity as well as immunogenicity: a novel strategy for modern vaccine development and a preliminary investigation about B cell discrimination of monomeric proteins. Eur J Immunol 35, 505–514 (2005). 15627976 10.1002/eji.200425749

[b62] Fleury A. *et al.* Detection of HP10 antigen in serum for diagnosis and follow-up of subarachnoidal and intraventricular human neurocysticercosis. J Neurol Neurosurg Psychiatry 78, 970–974 (2007). 17337467 10.1136/jnnp.2006.107243 PMC2117888

[b63] Zimic M. *et al.* Utility of a protein fraction with cathepsin L-Like activity purified from cysticercus fluid of *Taenia solium* in the diagnosis of human cysticercosis. Am J Trop Med Hyg 80, 964–970 (2009). 19478259 PMC2762273

[b64] Sciutto E. *et al.* Diagnosis of porcine cysticercosis: a comparative study of serological tests for detection of circulating antibody and viable parasites. Vet Parasitol 78, 185–194 (1998). 9760060 10.1016/s0304-4017(98)00129-0

[b65] Gotz S. *et al.* B2G-FAR, a species-centered GO annotation repository. Bioinformatics 27, 919–924 (2011). 21335611 10.1093/bioinformatics/btr059 PMC3065692

[b66] Gotz S. *et al.* High-throughput functional annotation and data mining with the Blast2GO suite. Nucleic Acids Res 36, 3420–3435 (2008). 18445632 10.1093/nar/gkn176 PMC2425479

