# Erratum: Genome analysis of Excretory/Secretory proteins in *Taenia solium* reveals their Abundance of Antigenic Regions (AAR)

**DOI:** 10.1038/srep12385

**Published:** 2015-09-16

**Authors:** Sandra Gomez, Laura Adalid-Peralta, Hector Palafox-Fonseca, Vito Adrian Cantu-Robles, Xavier Soberón, Edda Sciutto, Gladis Fragoso, Raúl J. Bobes, Juan P. Laclette, Luis del Pozo Yauner, Adrián Ochoa-Leyva

Scientific Reports 5: Article number: 968310.1038/srep09683; published online: 05192015; updated: 09162015

The original version of this Article incorrectly listed the current address of Adrián Ochoa-Leyva as ‘Department of Molecular Biophysics and Biochemistry, Yale University, 266 Whitney Avenue, New Haven, CT 06520-8114, USA.’ The correct address is listed below:

Departamento de Microbiología Molecular, Instituto de Biotecnología, Universidad Nacional Autónoma de México, Avenida Universidad 2001, Cuernavaca, C.P. 62210, México.

This has now been corrected in the HTML and PDF versions of the Article.

