# Supplementary material for: Genome analysis of Excretory/Secretory proteins in Taenia solium reveals their Abundance of Antigenic Regions (AAR)
Source: Sci Rep. 2015 May 19;5:9683. doi: 10.1038/srep09683 (PMC4437048; doi:10.1038/srep09683)
Supplement: Supplementary Information [file srep09683-s1.pdf]

Supplementary information

Genome analysis of Excretory/Secretory proteins in *T. solium* genome and novel measurement for their Abundance of Antigenic Regions (AAR)

Sandra Gomez[1], Laura Adalid-Peralta[1,2], Hector Palafox-Fonseca[1], Vito Adrian Cantu-Robles[3], Xavier Soberón[3,4], Edda Sciutto[5], Gladis Fragoso[5], Raúl J Bobes [5], Juan P Laclette[5], Luis del Pozo Yauner[3] and Adrián Ochoa-Leyva[6*]

Table S1. Enriched GO terms (Cellular Component) in *T. solium* secretome

Ranking	GO-ID	Term	FDR
1	GO:0005576	extracellular region	1.44E-29
2	GO:0044421	extracellular region part	1.21E-09
3	GO:0031012	extracellular matrix	9.29E-08
4	GO:0005615	extracellular space	1.79E-06
5	GO:0005578	proteinaceous extracellular matrix	3.17E-06
6	GO:0005788	endoplasmic reticulum lumen	4.30E-05
7	GO:0031225	anchored component of membrane	7.58E-05
8	GO:0005581	collagen trimer	7.43E-04
9	GO:0044420	extracellular matrix part	1.71E-02
10	GO:0098644	complex of collagen trimers	2.18E-02
11	GO:0044432	endoplasmic reticulum part	3.37E-02
12	GO:0005783	endoplasmic reticulum	4.44E-02
13	GO:0009986	cell surface	4.44E-02
14	GO:0005589	collagen type VI trimer	4.44E-02
15	GO:0098647	collagen beaded filament	4.44E-02

Table S2. Enriched GO terms (Molecular Function) in *T. solium* secretome

Ranking	GO-ID	Term	FDR
1	GO:0061134	peptidase regulator activity	1.43E-17
2	GO:0030414	peptidase inhibitor activity	3.92E-16
3	GO:0004857	enzyme inhibitor activity	1.00E-10
4	GO:0004866	endopeptidase inhibitor activity	8.00E-09
5	GO:0061135	endopeptidase regulator activity	9.72E-09
6	GO:0004867	serine-type endopeptidase inhibitor activity	7.39E-07
7	GO:0016504	peptidase activator activity	7.53E-05
8	GO:0005201	extracellular matrix structural constituent	7.94E-05
9	GO:0016706	oxidoreductase activity, acting on paired donors, with incorporation or reduction of molecular oxygen, 2-oxoglutarate as one donor, and incorporation of one atom each of oxygen into both donors	4.45E-04
10	GO:0030234	enzyme regulator activity	1.20E-03
11	GO:0031418	L-ascorbic acid binding	2.56E-03
12	GO:0098772	molecular function regulator	2.94E-03
13	GO:0008233	peptidase activity	3.53E-03
14	GO:0019842	vitamin binding	1.71E-02
15	GO:0070815	peptidyl-lysine 5-dioxygenase activity	1.71E-02
16	GO:0031545	peptidyl-proline 4-dioxygenase activity	1.71E-02
17	GO:0031543	peptidyl-proline dioxygenase activity	1.71E-02
18	GO:0004656	procollagen-proline 4-dioxygenase activity	1.71E-02
19	GO:0008475	procollagen-lysine 5-dioxygenase activity	1.71E-02
20	GO:0019798	procollagen-proline dioxygenase activity	1.71E-02

21	GO:0051213	dioxygenase activity	2.08E-02
22	GO:0016705	oxidoreductase activity, acting on paired donors, with incorporation or reduction of molecular oxygen	2.71E-02
23	GO:0004553	hydrolase activity, hydrolyzing O-glycosyl compounds	3.64E-02
24	GO:0070011	peptidase activity, acting on L-amino acid peptides	3.76E-02
25	GO:0030246	carbohydrate binding	3.86E-02
26	GO:0016702	oxidoreductase activity, acting on single donors with incorporation of molecular oxygen, incorporation of two atoms of oxygen	4.44E-02
27	GO:0016701	oxidoreductase activity, acting on single donors with incorporation of molecular oxygen	4.44E-02
28	GO:0005509	calcium ion binding	4.44E-02
29	GO:0051425	PTB domain binding	4.44E-02
30	GO:0003990	acetylcholinesterase activity	4.44E-02
31	GO:0004035	alkaline phosphatase activity	4.44E-02
32	GO:0016798	hydrolase activity, acting on glycosyl bonds	4.85E-02
33	GO:0003756	protein disulfide isomerase activity	4.85E-02

Table S3. Enriched GO terms (Biological Process) in *T. solium* secretome

Ranking	GO-ID	Term	FDR
1	GO:0052547	regulation of peptidase activity	2.63E-08
2	GO:0010466	negative regulation of peptidase activity	1.31E-07
3	GO:0051346	negative regulation of hydrolase activity	1.57E-06
4	GO:0006508	proteolysis	6.65E-06
5	GO:0030198	extracellular matrix organization	1.27E-05
6	GO:0045861	negative regulation of proteolysis	3.53E-05
7	GO:0010955	negative regulation of protein processing	4.26E-05
8	GO:0016485	protein processing	4.30E-05
9	GO:0070613	regulation of protein processing	4.63E-05
10	GO:0043062	extracellular structure organization	9.72E-05
11	GO:0030162	regulation of proteolysis	5.83E-04
12	GO:0010952	positive regulation of peptidase activity	6.52E-03
13	GO:0006917	apoptotic process	1.40E-02
14	GO:0007155	cell adhesion	1.71E-02
15	GO:0022610	biological adhesion	1.71E-02
16	GO:0019471	4-hydroxyproline metabolic process	1.71E-02
17	GO:0018401	peptidyl-proline hydroxylation to 4-hydroxy-L-proline	1.71E-02
18	GO:0018126	protein hydroxylation	1.71E-02
19	GO:0051563	smooth endoplasmic reticulum calcium ion homeostasis	1.71E-02
20	GO:0048669	collateral sprouting in absence of injury	1.71E-02
21	GO:0048668	collateral sprouting	1.71E-02
22	GO:0019511	peptidyl-proline hydroxylation	1.71E-02
23	GO:0043086	negative regulation of catalytic activity	2.25E-02
24	GO:0051248	negative regulation of protein metabolic process	3.76E-02
25	GO:0051336	regulation of hydrolase activity	3.96E-02
26	GO:0000904	cell morphogenesis involved in differentiation	4.13E-02
27	GO:0032269	negative regulation of cellular protein metabolic process	4.44E-02
28	GO:0032989	cellular component morphogenesis	4.44E-02
29	GO:0006581	acetylcholine catabolic process	4.44E-02
30	GO:0032469	endoplasmic reticulum calcium ion homeostasis	4.44E-02
31	GO:0016199	axon midline choice point recognition	4.85E-02
32	GO:0016198	axon choice point recognition	4.85E-02

Table S4. KEGG pathways in *T. solium* secretome

Ranking	KO	Kegg Pathway	Number of proteins
**1**	ko04141	Protein processing in endoplasmic reticulum	11
**2**	ko04142	Lysosome	10
**3**	ko05200	Pathways in cancer	10
**4**	ko04510	Focal adhesion	9
**5**	ko04390	Hippo signaling pathway	7
**6**	ko05205	Proteoglycans in cancer	7
**7**	ko00230	Purine metabolism	5
**8**	ko04310	Wnt signaling pathway	5
**9**	ko04151	PI3K-Akt signaling pathway	5
**10**	ko04145	Phagosome	5
**11**	ko04974	Protein digestion and absorption	5
**12**	ko05034	Alcoholism	5
**13**	ko05169	Epstein-Barr virus infection	5
**14**	ko00564	Glycerophospholipid metabolism	4
**15**	ko00240	Pyrimidine metabolism	4
**16**	ko00514	Other types of O-glycan biosynthesis	4
**17**	ko00511	Other glycan degradation	4
**18**	ko03020	RNA polymerase	4
**19**	ko04350	TGF-beta signaling pathway	4
**20**	ko04080	Neuroactive ligand-receptor interaction	4
**21**	ko04512	ECM-receptor interaction	4
**22**	ko04810	Regulation of actin cytoskeleton	4
**23**	ko04612	Antigen processing and presentation	4
**24**	ko04728	Dopaminergic synapse	4
**25**	ko04713	Circadian entrainment	4
**26**	ko05202	Transcriptional misregulation in cancer	4
**27**	ko05222	Small cell lung cancer	4
**28**	ko05010	Alzheimer's disease	4
**29**	ko05030	Cocaine addiction	4
**30**	ko05031	Amphetamine addiction	4
**31**	ko05166	HTLV-I infection	4
**32**	ko05146	Amoebiasis	4
**33**	ko01110	Biosynthesis of secondary metabolites	3
**34**	ko03018	RNA degradation	3
**35**	ko04014	Ras signaling pathway	3
**36**	ko04015	Rap1 signaling pathway	3
**37**	ko04010	MAPK signaling pathway	3
**38**	ko04340	Hedgehog signaling pathway	3

**39**	ko04391	Hippo signaling pathway - fly	3
**40**	ko04020	Calcium signaling pathway	3
**41**	ko04623	Cytosolic DNA-sensing pathway	3
**42**	ko04670	Leukocyte transendothelial migration	3
**43**	ko04261	Adrenergic signaling in cardiomyocytes	3
**44**	ko04270	Vascular smooth muscle contraction	3
**45**	ko04724	Glutamatergic synapse	3
**46**	ko04720	Long-term potentiation	3
**47**	ko04721	Synaptic vesicle cycle	3
**48**	ko04360	Axon guidance	3
**49**	ko05217	Basal cell carcinoma	3
**50**	ko05016	Huntington's disease	3
**51**	ko05410	Hypertrophic cardiomyopathy (HCM)	3
**52**	ko05412	Arrhythmogenic right ventricular cardiomyopathy (ARCV)	3
**53**	ko05414	Dilated cardiomyopathy	3
**54**	ko05152	Tuberculosis	3
**55**	ko01212	Fatty acid metabolism	2
**56**	ko00310	Lysine degradation	2
**57**	ko00480	Glutathione metabolism	2
**58**	ko00563	Glycosylphosphatidylinositol (GPI)-anchor biosynthesis	2
**59**	ko00860	Porphyrin and chlorophyll metabolism	2
**60**	ko03060	Protein export	2
**61**	ko04120	Ubiquitin mediated proteolysis	2
**62**	ko04012	ErbB signaling pathway	2
**63**	ko04330	Notch signaling pathway	2
**64**	ko04144	Endocytosis	2
**65**	ko04111	Cell cycle - yeast	2
**66**	ko04113	Meiosis - yeast	2
**67**	ko04520	Adherens junction	2
**68**	ko04530	Tight junction	2
**69**	ko04610	Complement and coagulation cascades	2
**70**	ko04910	Insulin signaling pathway	2
**71**	ko04914	Progesterone-mediated oocyte maturation	2
**72**	ko04916	Melanogenesis	2
**73**	ko04260	Cardiac muscle contraction	2
**74**	ko04972	Pancreatic secretion	2
**75**	ko04726	Serotonergic synapse	2
**76**	ko04722	Neurotrophin signaling pathway	2
**77**	ko05206	MicroRNAs in cancer	2
**78**	ko05210	Colorectal cancer	2

**79**	ko05322	Systemic lupus erythematosus	2
**80**	ko05323	Rheumatoid arthritis	2
**81**	ko05014	Amyotrophic lateral sclerosis (ALS)	2
**82**	ko05033	Nicotine addiction	2
**83**	ko05110	Vibrio cholerae infection	2
**84**	ko05120	Epithelial cell signaling in Helicobacter pylori infection	2
**85**	ko05130	Pathogenic Escherichia coli infection	2
**86**	ko05132	Salmonella infection	2
**87**	ko05131	Shigellosis	2
**88**	ko05100	Bacterial invasion of epithelial cells	2
**89**	ko05164	Influenza A	2
**90**	ko05161	Hepatitis B	2
**91**	ko05168	Herpes simplex infection	2
**92**	ko05145	Toxoplasmosis	2
**93**	ko05142	Chagas disease (American trypanosomiasis)	2
**94**	ko01120	Microbial metabolism in diverse environments	1
**95**	ko00052	Galactose metabolism	1
**96**	ko00500	Starch and sucrose metabolism	1
**97**	ko00520	Amino sugar and nucleotide sugar metabolism	1
**98**	ko00190	Oxidative phosphorylation	1
**99**	ko00910	Nitrogen metabolism	1
**100**	ko00062	Fatty acid elongation	1
**101**	ko00071	Fatty acid degradation	1
**102**	ko00100	Steroid biosynthesis	1
**103**	ko00565	Ether lipid metabolism	1
**104**	ko00590	Arachidonic acid metabolism	1
**105**	ko01040	Biosynthesis of unsaturated fatty acids	1
**106**	ko00280	Valine, leucine and isoleucine degradation	1
**107**	ko00330	Arginine and proline metabolism	1
**108**	ko00430	Taurine and hypotaurine metabolism	1
**109**	ko00460	Cyanoamino acid metabolism	1
**110**	ko00510	N-Glycan biosynthesis	1
**111**	ko00533	Glycosaminoglycan biosynthesis - keratan sulfate	1
**112**	ko00531	Glycosaminoglycan degradation	1
**113**	ko00601	Glycosphingolipid biosynthesis - lacto and neolacto series	1
**114**	ko00603	Glycosphingolipid biosynthesis - globo series	1

**115**	ko00604	Glycosphingolipid biosynthesis - ganglio series	1
**116**	ko00785	Lipoic acid metabolism	1
**117**	ko00790	Folate biosynthesis	1
**118**	ko00830	Retinol metabolism	1
**119**	ko00627	Aminobenzoate degradation	1
**120**	ko03022	Basal transcription factors	1
**121**	ko03040	Spliceosome	1
**122**	ko03015	mRNA surveillance pathway	1
**123**	ko04130	SNARE interactions in vesicular transport	1
**124**	ko03410	Base excision repair	1
**125**	ko03420	Nucleotide excision repair	1
**126**	ko02020	Two-component system	1
**127**	ko04370	VEGF signaling pathway	1
**128**	ko04668	TNF signaling pathway	1
**129**	ko04514	Cell adhesion molecules (CAMs)	1
**130**	ko04140	Regulation of autophagy	1
**131**	ko04110	Cell cycle	1
**132**	ko04114	Oocyte meiosis	1
**133**	ko04115	p53 signaling pathway	1
**134**	ko04540	Gap junction	1
**135**	ko04620	Toll-like receptor signaling pathway	1
**136**	ko04621	NOD-like receptor signaling pathway	1
**137**	ko04622	RIG-I-like receptor signaling pathway	1
**138**	ko04664	Fc epsilon RI signaling pathway	1
**139**	ko04062	Chemokine signaling pathway	1
**140**	ko04911	Insulin secretion	1
**141**	ko04920	Adipocytokine signaling pathway	1
**142**	ko03320	PPAR signaling pathway	1
**143**	ko04912	GnRH signaling pathway	1
**144**	ko04917	Prolactin signaling pathway	1
**145**	ko04918	Thyroid hormone synthesis	1
**146**	ko04970	Salivary secretion	1
**147**	ko04971	Gastric acid secretion	1
**148**	ko04978	Mineral absorption	1
**149**	ko04966	Collecting duct acid secretion	1
**150**	ko04725	Cholinergic synapse	1
**151**	ko04730	Long-term depression	1
**152**	ko04723	Retrograde endocannabinoid signaling	1
**153**	ko04745	Phototransduction - fly	1
**154**	ko04740	Olfactory transduction	1
**155**	ko04380	Osteoclast differentiation	1
**156**	ko05212	Pancreatic cancer	1

**157**	ko05219	Bladder cancer	1
**158**	ko05012	Parkinson's disease	1
**159**	ko05020	Prion diseases	1
**160**	ko05416	Viral myocarditis	1
**161**	ko04940	Type I diabetes mellitus	1
**162**	ko04930	Type II diabetes mellitus	1
**163**	ko04932	Non-alcoholic fatty liver disease (NAFLD)	1
**164**	ko04950	Maturity onset diabetes of the young	1
**165**	ko05133	Pertussis	1
**166**	ko05160	Hepatitis C	1

Table S5. Set of proteins experimentally used in the diagnosis of human *T. solium* infection

GI identifier	Sequence name	E-value (from BLASTP against 838 ES proteins)	RNA support
452851	antigen B [Echinococcus granulosus]	2.00E-10	Yes
452853	antigen B [Echinococcus multilocularis]	4.00E-06	Yes
4960053	AF147955_1 antigen cC1 [Taenia solium]	No hits found	Not determined
5802763	AF098074_1 18 kDa glycoprotein TS18 variant 2 precursor [Taenia solium]	9.00E-37	Yes
6288722	cysticercosis-specific antigen [Taenia solium]	4.00E-31	Yes
7339851	immunogenic protein Ts21 [Taenia solium]	5.00E-34	Yes
9963978	AF249884_1 hydrophobic ligand binding protein [Hymenolepis diminuta]	9.00E-11	Yes
11527691	low molecular weight antigen 1 [Taenia solium]	9.00E-37	Yes
11527695	low molecular weight antigen 2 [Taenia solium]	2.00E-35	Yes
11527697	low molecular weight antigen 2 variant 1 [Taenia solium]	3.00E-33	Yes
11935118	hydrophobic ligand binding protein [Moniezia expansa]	3.00E-09	Yes
14495278	AF361934_1 AgB subunit [Echinococcus granulosus]	3.00E-12	Yes
19879948	AF356337_1 8 kDa diagnostic antigen Ts14 variant 1 [Taenia solium]	7.00E-34	Yes
19879950	AF356338_1 8 kDa diagnostic antigen Ts14 variant 2 [Taenia solium]	4.00E-34	Yes
19879954	AF356340_1 8 kDa diagnostic antigen TsRS1 variant 1 [Taenia solium]	6.00E-39	Yes
19879958	AF356342_1 8 kDa diagnostic antigen TsRS1 variant 2, partial [Taenia solium]	2.00E-39	Yes
19879962	AF356344_1 8 kDa diagnostic antigen TsRS2, partial [Taenia solium]	9.00E-40	Yes
21930119	AF523312_1 oncosphere-specific antigen [Taenia solium]	1.00E-25	Yes
22657785	AF082828_1 18 kDa glycoprotein TS18 precursor [Taenia solium]	7.00E-39	Yes
22657787	AF082830_1 RS1 [Taenia solium]	2.00E-34	Yes

22657791	AF098073_1 18 kDa glycoprotein TS18 variant 1 precursor [Taenia solium]	5.00E-34	Yes
22760506	AF082829_1 14 kDa glycoprotein TS14 precursor [Taenia solium]	7.00E-39	Yes
23477222	secreted antigen Ts8B1 [Taenia solium]	1.00E-26	Yes
23477224	secreted antigen Ts8B2 [Taenia solium]	2.00E-34	Yes
23477226	secreted antigen Ts8B3 [Taenia solium]	1.00E-19	Yes
27808486	antigen B [Echinococcus multilocularis]	9.00E-08	Yes
30523226	antigen B subunit 1 [Echinococcus oligarthrus]	3.00E-07	Yes
32130571	antigen B subunit 1 [Echinococcus vogeli]	4.00E-09	Yes
37787347	diagnostic antigen GP50b precursor [Taenia solium]	3.00E-161	Yes
74766798	Q26863_9CEST Immunodiagnostic antigen	2.00E-30	Yes
74811268	Q7YW44_TAESO Glucoprotein	4.00E-37	Yes
74812179	Q867W2_ECHMU Antigen B8/1b (Antigen B)	1.00E-07	Yes
74819408	Q8T7K5_TAESO 8 kDa diagnostic antigen TsRS2 variant 1	2.00E-41	Yes
74823170	Q9BIV3_TAESO 18 kDa glycoprotein variant 1	4.00E-46	Yes
74835275	Q27275_ECHGR Antigen B 8 kDa subunit precursor (Antigen B subunit 2 precursor) (Antigen B subunit 2) (Antigen B precursor)	2.00E-09	Yes
74837032	Q5FAR4_ECHMU Antigen B	2.00E-26	Yes
74837033	Q5FAR5_ECHMU Antigen B	2.00E-11	Yes
74849582	Q9U562_TAESO 18 kDa glycoprotein TS18 variant 3 precursor	1.00E-37	Yes
112819967	M13h variant [Taenia solium]	3.00E-33	Yes
112819969	B1 variant [Taenia solium]	9.00E-32	Yes
122063423	Q6Q0G7_ECHGR Antigen B subunit 4	2.00E-05	Yes
122103070	Q0GH68_TAESO 14 kDa glycoprotein TS14	4.00E-38	Yes
188485737	Nc-DigChim-324430 [synthetic construct]	2.00E-33	Yes
261266611	enolase [Taenia asiatica]	No hits found	Not determined

311335041	trypsin-like protein [Taenia solium]	0	Yes
347546145	CyDA variant 1 [Taenia solium]	7.00E-34	Yes
